# Mapping the global research landscape and knowledge gaps on multimorbidity: a bibliometric study

**DOI:** 10.7189/jogh.07.010414

**Published:** 2017-06

**Authors:** Xiaolin Xu, Gita D Mishra, Mark Jones

**Affiliations:** Centre for Longitudinal and Life Course Research, School of Public Health, The University of Queensland, Brisbane, Australia

## Abstract

**Background:**

To summarize global research trends and activities on multimorbidity; then to assess the knowledge gaps and to identify implications for knowledge exchange between high income countries (HICs) and low– and middle– income countries (LMICs).

**Methods:**

A comprehensive search was conducted to identify research publications on multimorbidity in the Web of Science^TM^, as well as diabetes, depression, hypertension, and Chronic Obstructive Pulmonary Disease (COPD). The time frame for the search was from 1900 to June, 2016. Information (such as publication date, subject category, author, country of origin, title, abstract, and keywords) were extracted and the full texts were obtained for the co–citation analysis. Data were linked with the life expectancy at birth (years) and Gross National Income (GNI). Co–citation and hierarchal clustering analysis was used to map the trends and research networks with CiteSpace II (JAVA freeware, copyright Chaomei Chen, http://cluster.cis.drexel.edu/~cchen/citespace/).

**Findings:**

We identified 2864 relevant publications as at June 2016, with the first paper on this topic indexed in 1974 from Germany, but 80% were published after 2010. Further analysis yielded two knowledge gaps: (1) compared with single conditions (diabetes, hypertension, depression, and COPD), there is a mismatch between the high prevalence of multimorbidity and its research outputs (ratio of articles on multimorbidity vs other four single conditions is 1:13–150); (2) although a total of 76 countries have contributed to this research area, only 5% of research originated from LMICs where 73% of non–communicable disease (NCD) related deaths had occurred. Additional analysis showed the median year of first publication occurred 15 years later in the LMICs compared with HICs (2010 vs 1995); and longer life expectancy was associated with exponentially higher publication outputs (Pearson correlation coefficient *r* = 0.95) at the global level. The life expectancy at the median year (1994) of first publication was 66.1, with the gap between LMICs and HICs 7.9 (68.4 vs 76.3).

**Conclusions:**

This study confirms substantial knowledge gaps in the research agenda on multimorbidity, with input urgently needed to move us forward worldwide, especially for and in LMICs. There is the possibility that LMICs can learn from and collaborate with HICs in this area.

Noncommunicable diseases (NCDs) have accounted for 68% of the world’s 56 million deaths in 2012 [1], with half of the people with NCDs having two or more conditions (multimorbidity) [2,3]. Multimorbidity is a comparatively new concept and a challenging area in medical practice globally. Although it is simply defined as “the coexistence of multiple chronic conditions in a given individual” [4,5], based on the research to date [2,3], there is no consensus on which conditions should be considered; or on the method used for measuring multimorbidity. This makes comparisons between– or among studies difficult.

With increasing population and life expectancy, the disease burden of multimorbidity to both individuals and society are increasing. Multimorbidity is already, and will be in the future, a great challenge for both developed– and developing country settings [3,5]. A staggering toll of about 80% of NCDs deaths occurred in low income settings [6], and the most socioeconomically deprived areas have substantially more multimorbidity, that happens earlier (10–15 years) than do their most affluent peers [2], however, evidence from low income settings is limited [3,7-9]. The identification and implementation of innovative approaches are essential for tackling this growing epidemic, especially for and in LMICs.

Better understanding of the whole picture of global research trends, activities and identification of the knowledge gaps on multimorbidity is necessary to move the research agenda forward, and especially for LMICs. This has coincided with the World Health Organization (WHO) global health and research priority on healthy aging [3,10]. While some studies have pointed to the mismatch between the importance of multimorbidity and research outputs in this area [11–13], only a few studies have comprehensively evaluated the research trends, knowledge gaps, and inequality among countries. We aimed to use a comprehensive bibliometric analysis to document these gaps at a global level.

## METHODS

### Data sources and search strategy

A comprehensive search was conducted to identify research publications on multimorbidity including its various spellings (multiple chronic diseases, multiple chronic conditions, polymorbidity, polypathology, pluripathology, and multipathology) in the Web of Science^TM^, Core Collection database, one of the world largest databases of peer–reviewed literature provided by the Thomson Scientific Institute. The time frame for the search was from 1900 to June, 2016. Detailed search strategies can be found in Appendix S1 in **Online Supplementary Document(Online Supplementary Document)**. The same search strategies were performed on four other leading causes of death: diabetes, depression, hypertension, and COPD. We included all publication types (such as, article, meeting abstracts, review, editorial, letter, etc.).

We also collected the life expectancy at birth (years) and gross national income (GNI) per capita from the website of the World Bank to examine the relationship between these variables and research outputs. The income groups included low–income (US$ 1025 or less), lower middle–income (US$ 1026 to US$ 4035), upper middle–income (US$ 4036 to US$ 12 475), and high–income (US$ 12 476 or more) economies based on per capita gross national income.

### Data analysis and visualization

All publications were included with the following variables extracted: publication date, subject category, document type, author, organization of origin, funding agency, language, country of origin, title, abstract, and keyword. In addition, the full texts were obtained for the co–citation analysis. The retrieved results were exported to both Microsoft Office Excel (Microsoft, Seattle, WA, USA) and plain text for further analysis.

Data were linked with the life expectancy at birth (years) and GNI in Microsoft Office Excel. The Pearson correlation coefficient (*r*) between life expectancy at birth (year) and annual publications (on the logarithmic scale) was calculated.

CiteSpace II [14] was used to conduct co–citation and hierarchal clustering analysis to map the trends and research networks.

### Ethical issues

The research used published data from secondary sources and did not involve any interactions with human subjects. Hence it is exempt from the institutional review board (IRB) approval process.

## RESULTS

### Characteristics of research landscapes on multimorbidity worldwide

**Figure 1:**
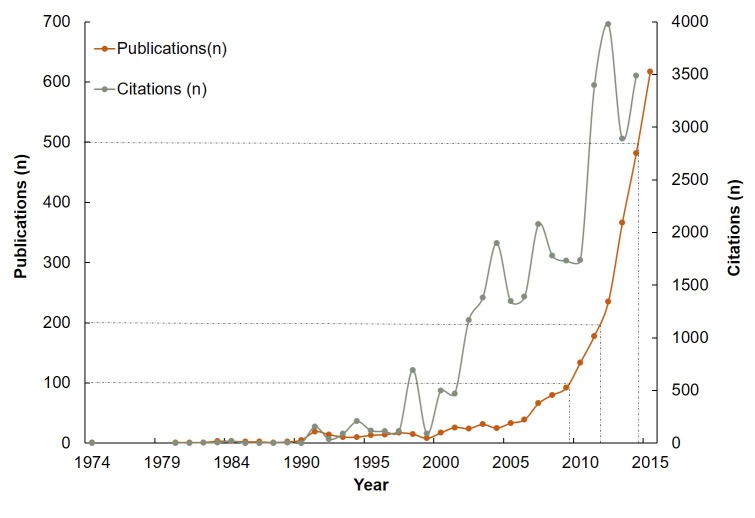
Trends of the annual publications and citations on multimorbidity worldwide.

A total of 2864 articles on multimorbidity were retrieved from the database, with the first indexed in 1974 from Germany. As shown in **Figure 1**, the publications appeared sporadically before 1990, and increased slowly up to the early 2000s, with a transition to exponential growth after 2005. Of the 2864 articles, 80% were published after 2010, while only 9% appeared between 1974 and 2004. Regarding the development of annual total citations, **Figure 1** shows a similar trajectory to the publication data with a total of 31 669 citations. However, the exponential growth started around 2000.

The primary type of publication was research article (74%) (see Appendix S2 in **Online Supplementary Document(Online Supplementary Document)**). There were 12 publication languages, 87% were reported in English, followed by German (10%), Spanish (1.3%) and French (1.0%) (see Appendix S3 in **Online Supplementary Document(Online Supplementary Document)**). The publications covered 897 journals, and we identified the top 20 journals had published around or more than 20 articles between 1974 and 2016. Journals with the most articles on multimorbidity include the *Journal of the American Geriatrics Society* (n = 74), *PLOS One* (n = 74) and *BMC Family Practice* (n = 67). Further details on the top 20 journals can be found in Appendix S4 in **Online Supplementary Document(Online Supplementary Document)**. Of the 20 most prolific authors, 15 were from Europe, four were from North America, and one was from Australia, with none from LMICs (see Appendix S5 in **Online Supplementary Document(Online Supplementary Document)**).

Overall, the three leading research institutions (Johns Hopkins University, Harvard University, and University of California System) were from the US, followed by Germany and UK institutions (see Appendix S6 in **Online Supplementary Document(Online Supplementary Document)**). Visualization of the institutions performance and collaborative networks can be seen in Appendix S7 in **Online Supplementary Document(Online Supplementary Document)**. Nine of the top 15 funding agencies from US sponsored the most publications (see Appendix S8 in **Online Supplementary Document(Online Supplementary Document)**).

A total of 117 different subject categories were involved in this research area, with the leading three subject categories: Medicine General Internal (691 papers), Health Care Sciences Services (422 papers), and Geriatrics Gerontology (381 papers). The top 20 subject categories are listed in Appendix S9 in **Online Supplementary Document(Online Supplementary Document)**. The different subject categories connected broadly, imply that multimorbidity research is an interdisciplinary area (see Appendix S10 in **Online Supplementary Document(Online Supplementary Document)**).

### Knowledge Gap 1. Mismatch between the high prevalence of multimorbidity and its publication outputs

Compared with other high prevalence single chronic condition (diabetes, hypertension, depression, and COPD), there is a mismatch between the high prevalence of multimorbidity and its research outputs. Although the median prevalence of multimorbidity is 63%, the number of articles on the other four single conditions is 13 to 150 times that of multimorbidity (**Table 1**).

**Table 1:** Prevalence of certain conditions and number of related articles published to date

Conditions	Prevalence (year)	Publications	Ratio of articles on multimorbidity vs on other four conditions
Multimorbidity	13%–83% (1989–2012) [15]*	2864	1:1
Diabetes	9% (2014) [1]	431 009	1:150
Depression	5.9–14.6 (2000s) [16]	360 666	1:126
Hypertension	22% (2014) [1]	346 894	1:121
COPD	11.7% (2010) [17]	36 866	1:13

*A systematic review included articles from 1989 to 2012, with median prevalence 63%.

Further analysis demonstrated both physical and mental disorders are contained in the research framework of multimorbidity. **Table 2** shows the top 10 featured conditions and leading risk factors mentioned in the keywords list. Similar findings were also found in other research types, such as systematic review [18], and longitudinal cohort study [19]. The leading disease and risk factor was cardiovascular and heart disease, and physical activity, respectively.

**Table 2:** Featured conditions and risk factors mentioned in the keywords list

Rank	Conditions or risk factors	Appearance (n)
**Diseases:**		
1	Cardiovascular and heart disease	340
2	Depression	195
3	Diabetes mellitus	94
4	Dementia	85
5	COPD	78
6	Cancer	70
7	Hypertension	56
8	Alzheimer disease	41
9	Chronic kidney disease	34
10	Cognitive impairment	33
**Risk factors:**		
1	Physical activity	51
2	Obesity	45
3	Body mass index	30

### Knowledge Gap 2. Imbalance in publications among countries and the roles of economics and life expectancy

**Table 3** outlines indicators to demonstrate the imbalance in research outputs on multimorbidity and NCD disease burden and life expectancy. Compared with 73% NCD related deaths that occurred in LMICs, only 5% of publications on multimorbidity originated from LMICs. Onset of the median year of first publication occurred 15 years later in the LMICs compared with HICs (2010 vs 1995.), and at that time point, the life expectancy at birth (years) was 66.1, with the gap between LMICs and HICs 7.9 (68.4 vs 76.3).

**Table 3:** Research outputs on multimorbidity vs NCD deaths and life expectancy in LMICs and HICs, 1974–2016

Indicators	Income group	
**LMICs**	**HICs**
Multimorbidity publications (%)	176 (5)	3510 (95)
NCD deaths (%)*	27 733 (73)	10 159 (27)
Median year of the first publication	2010	1995
Life expectancy at birth (years) at the median year of the first publication	68.4	76.3

LMIC – low– and middle–income countries, HIC – high–income countries

*Data sources: World Health Organization [20].

**Figure 2:**
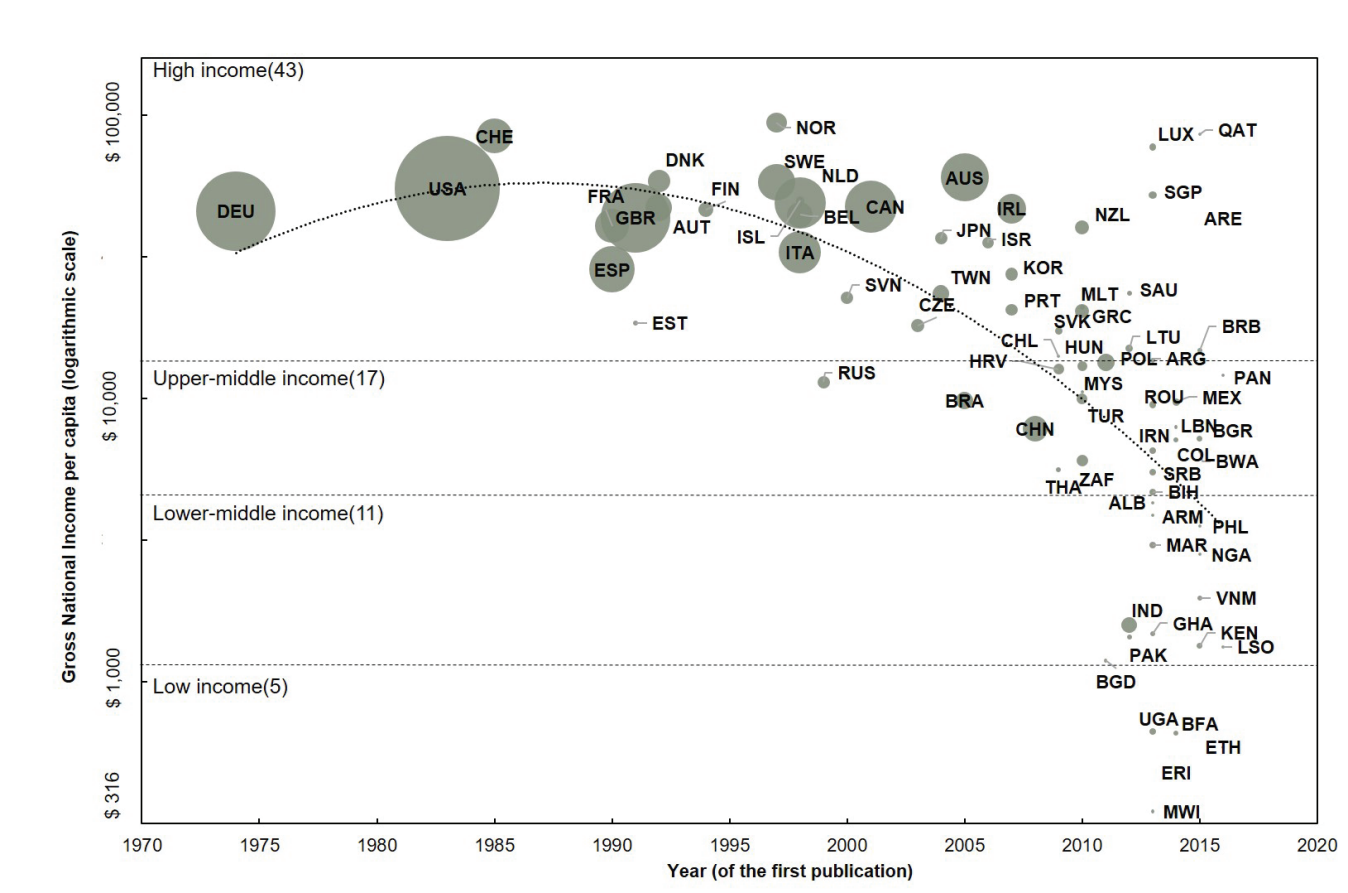
Publications distribution and changes in high, middle, and low income countries, 1974–2016. Marker size is proportional to the total publications between 1990 and 2016. The full results of the above figure can be found in Appendices S11 and S12 in **Online Supplementary Document(Online Supplementary Document).**

We analyzed the year of first publication and total number of publications by country. **Figure 2** shows the distributions and trends of publications between 1974 and 2016 plotted according to the level of GNI per capita in 2015 for the 76 countries with at least one publication indexed in the Web of Science^TM^. The GNI data sources for **Figure 2** are from the World Bank [21]. The chart illustrates the substantial knowledge gaps with a direct gradient apparent by income country status, especially compared with the disease burden (**Table 3**).

Among the income groups, there is a great deal of heterogeneity in the year of first publication and total number of publications. The number of countries in high income, upper–middle income, lower–middle income, and low–income countries is 43, 17, 11, and 5 respectively. Although Germany published earlier (1974), US holds the most publications (n = 895), followed by Germany (n = 511) and UK (n = 389). All the top 15 countries were HICs; the first LMIC being China with 52 publications, ranked 16th. The median year of the first publication in high income, upper–middle income, lower–middle income, and low income countries was 1995, 2010, 2013, and 2014 respectively. The first LMIC publication was in 2005 by Brazil, which is ranked 25th.

**Figure 3:**
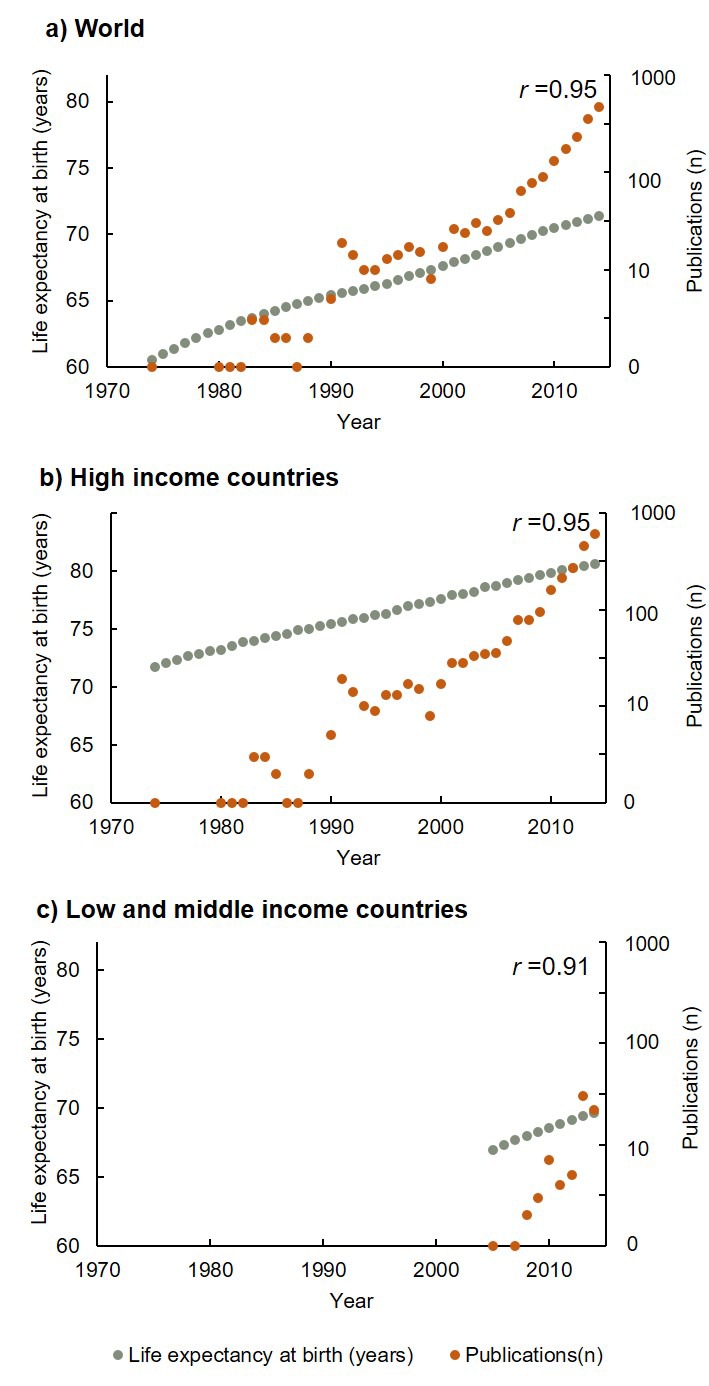
The relationship between life expectancy at birth (years) and annual publications (logarithmic scale), 1974–2014.

**Figure 3** shows the relationship between life expectancy at birth (years) and annual publications (on the logarithmic scale) from 1974 through 2014. Longer life expectancy was associated with higher publication outputs in both HICs and LMICs (*r* = 0.95 and 0.91, respectively). The life expectancy at the median year (1994) of first publication was 66.1, with a gap between LMICs and HICs of 7.9 (68.4 vs 76.3). The data sources for **Figure 3** are from the World Bank [22].

### Applications: research topics evolution over time and potential collaborations between HICs and LMICs

**Figure 4:**
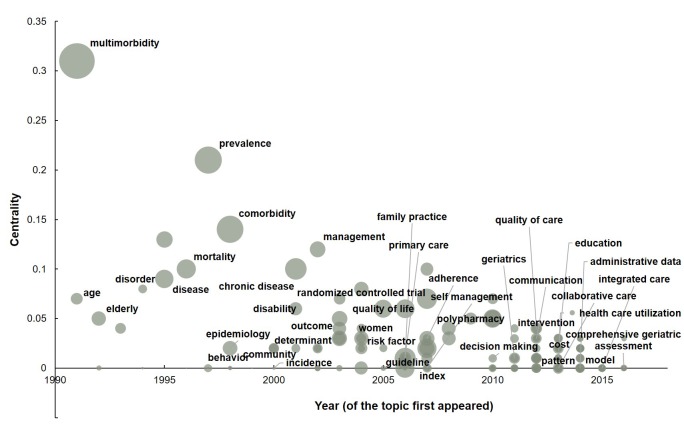
Research topics evolution on multimorbidity, 1990–2016. A node of high centrality is usually one that connects two or more large groups of nodes with the node itself in–between [14]. Centrality scores are normalized to the unit interval of [0, 1]. Marker size is proportional to number of times the keyword appeared between 1990 and 2016.

Terms from keywords were used to explore the emerging research trends and evolution topics [14]. 152 distinct topics from keywords of publications were obtained between 1990 and 2016 to illustrate the rapidly advancing research area. **Figure 4** shows the research topics evolution over the years, with topics beginning with depicting epidemiology characteristics (such as “prevalence”, “age”, “disease”, “disorders”, “chronic disease” and “mortality”), then moving to exploring risk factors, the impact of multimorbidty on individuals and the health system (such as “determinants”, “quality of life”, “family practice”, “self management”, “guideline”, “polypharmacy”, and “adherence”), and more recently examining interventions and how to improve the management (such as “intervention”, “education”, “comprehensive geriatric”, “collaborative and integrated care”).

**Figure 5:**
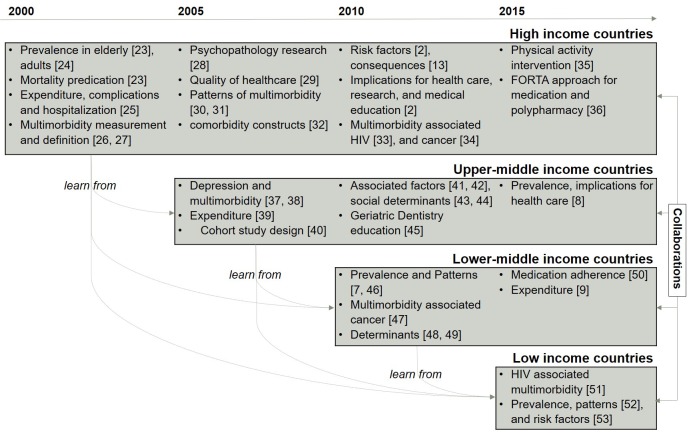
Featured research topics to illustrate the knowledge gaps and potential collaboration among countries, 2010–2016.

**Figure 5** shows the featured research topics (based upon the top cited publications from each income group in each year) evolution in each income group between 2000 and 2016, in an attempt to illustrate the knowledge gaps and potential collaboration among countries (the full results of the featured publications and the top 15 cited publications can be found in Appendices S13 and S14 in **Online Supplementary Document(Online Supplementary Document)**). Featured research topics in **Figure 5** were based on the top cited publications from each income group in each year: high–income countries [2,13,23–36], upper–middle income countries [8,37–45], lower-middle income countries [7,9,46–50], and low–income countries [51–53].

Most of the research topics evolution was similar among high and low income countries over time, especially in the initial stage when investigation began in this research area. It can be seen from the chart that HICs have contributed more knowledge than LMICs. If we take an epidemiology study as an example, HICs have published studies on “how to define and measure multimorbidity”. This research can potentially be used by other income group countries and tailored to fit their own contexts and populations. Other examples such as “multimorbidity associated HIV and cancer”, “physical activity intervention”, and “approaches for medication and polypharmacy” that had been investigated by HICs have only just emerged as a research topic in other countries.

As the figure shows, although collaborations have existed, such as in the FINE study (Finland, Italy, Netherlands) [23], and WHO’s Study on global Aging and adult health (SAGE) (China, Ghana, India, Mexico, Russia and South Africa) [9], these collaborations occurred within the same income groups. There were no collaborations between LMICs and HICs, however, potential collaborations between HICs and LMICs are possible.

## DISCUSSION

According to the best of our knowledge, this is the first study to evaluate the knowledge gaps and the potential for collaborations on multimorbidity between LMICs and HICs. Although it is well recognized that multimorbidity is a neglected research topic, such as in clinical guidelines and in randomized controlled trails (RCTs) [54–59], two of our findings and one implication are largely new.

First, compared with single conditions (diabetes, hypertension, depression, and COPD), there is a mismatch between the high prevalence of multimorbidity and its research outputs at the global level. This finding was also identified by Fortin and co–workers in the Canadian population among people aged 55 to 74 years [11]. Research conducted by the Emerging Risk Factors Collaboration [60] examined 689 300 participants from 91 cohorts and concluded that increasing numbers of chronic conditions within individuals are multiplicatively associated with increased mortality risk. Facing this huge disease burden, there is only a limited evidence base on which to inform policy and practice for these urgent health care needed individuals [61].

Second, research knowledge on multimorbidity from LMICs is comparatively limited compared with HICs (5% vs 95%), whereas nearly 80% of NCD related deaths occurred in LMICs. However, during the past 5 years, the number of publications originating from LMICs has grown substantially. Most publications were from upper–middle income countries, especially Brazil and China. On further analysis, we found that economic status and life expectancy played important roles in this gap, with higher economic status and longer life expectancy positively associated with higher annual publication outputs and the median year of first publication. One large study from Scotland found that the onset of multimorbidity occurred 10–15 years earlier in people living in the most deprived areas compared with the most affluent [2]. This finding coincides with two interesting statistics from our study: (1) onset of the median year of first publication occurred 15 years later in the LMICs compared with HICs, and (2) the life expectancy at the median year of first publication in LMICs was 7.9 years lower than in HICs. The potential implications from these findings are that: people from lower socioeconomic settings are more vulnerable to multimorbidity; and multiple chronic conditions is especially debilitating in LMICs, where they are facing the double burden of communicable and non–communicable diseases where dealing with premature, infectious diseases and single conditions are already great challenges.

Higher income is associated with greater longevity, with differences in life expectancy across income groups increasing [62]. With life expectancy increasing over the world, especially in LMICs, it is estimated that 80% of older people will be living in LMICs by 2050. This leads to questions of whether we have accumulated enough knowledge at a pace that can meet this challenge, especially in LMICs, and what more can we do based on the latest knowledge.

Despite the paucity of studies indicating the lack of research capacity and funding, LMICs may benefit from the theoretical and practical experience of HICs in multimorbidity research and implementation. However, tremendous national and international effort is needed to address the knowledge gaps between countries and provide better evidence to inform medical and public health decisions needs. The barriers to implementation of knowledge and experience generated from HICs needs to be evaluated, with the purpose to develop better availability and affordability in multimorbidity control and prevention strategies and measures. Take integrated care as an example; although it can create efficiency gains and improve health outcomes [63], it may only apply to HICs with good health systems. For LMICs, challenges from capacity building, better quality services, and a stronger evidence base will be required for implementing integration of care [3,64], but this is feasible as demonstrated by some operational models and approaches, such as the WHO 25 × 25 strategy (a target of a 25% relative reduction in NCD mortality by 2025) and the Integrated Management of Adult and Adolescent Illness (IMAI) [65,66].

Our findings demonstrate significant gaps in multimorbidity research between HICs and LMICs with limited knowledge of etiology, epidemiology, patterns, progression, risk factors and efficacy and cost–effectiveness of different interventions [64,67]. Knowledge on polypharmacy is lacking, as most of the evidence generated from RCTs is of limited value to guide decisions about medication use by patients with multiple chronic diseases as the possible drug–to–drug, drug–to–disease, and disease–to–disease interactions remain unexamined [5].

### Limitations

Several limitations are associated with our comprehensive bibliometric study. A linguistic bias may exist with the restriction to publications in English language journals. Second, in order to perform a high quality bibliometric analysis, we only searched the Web of Science database, which doesn’t include non–SCI journals, such as the *Journal of Comorbidity*, where there may be many multimorbidity relevant publications. In addition, we have not provided a detailed analysis of the research topics, such as the prevalence and disease burden of multimorbidity in each country or income group. In order to move the research agenda forward, we suggest topic specific systematic reviews which cover more databases, a secondary analysis of data at the global level, and primary longitudinal cohort studies are needed.

## CONCLUSION

This study confirms substantial knowledge gaps in multimorbidity research and between HICs and LMICs countries, research agenda and inputs needed to move research forward worldwide, especially for and in LMICs. There is a possibility that LMICs can learn from and collaborate with HICs in this area.

## References

[1] World Health Organization. Global status report on noncommunicable diseases 2014. 2015. Available: http://www.who.int/nmh/publications/ncd-status-report-2014/en/. Accessed: 7 July 2016.

[2] Barnett K, Mercer SW, Norbury M, Watt G, Wyke S, Guthrie B (2012). Epidemiology of multimorbidity and implications for health care, research, and medical education: a cross-sectional study.. Lancet.

[3] World Health Organization. World Health Report on ageing and health. Available: http://apps.who.int/iris/bitstream/10665/186463/1/9789240694811_eng.pdf. Accessed: 7 July 2016.

[4] Mercer S, Salisbury C, Fortin M. ABC of multimorbidity. Hoboken; John Wiley & Sons; 2014.

[5] Parekh AK, Goodman RA, Gordon C, Koh HK (2011). HHS Interagency Workgroup on Multiple Chronic Conditions. Managing multiple chronic conditions: a strategic framework for improving health outcomes and quality of life.. Public Health Rep.

[6] Hunter DJ, Reddy KS (2013). Noncommunicable diseases.. N Engl J Med.

[7] Khanam MA, Streatfield PK, Kabir ZN, Qiu C, Cornelius C, Wahlin A (2011). Prevalence and patterns of multimorbidity among elderly people in rural Bangladesh: a cross-sectional study.. J Health Popul Nutr.

[8] Wang HH, Wang JJ, Wong SY, Wong MC, Li FJ, Wang PX (2014). Epidemiology of multimorbidity in China and implications for the healthcare system: cross-sectional survey among 162,464 community household residents in southern China.. BMC Med.

[9] Arokiasamy P, Uttamacharya U, Jain K, Biritwum RB, Yawson AE, Wu F (2015). The impact of multimorbidity on adult physical and mental health in low- and middle-income countries: what does the study on global ageing and adult health (SAGE) reveal?. BMC Med.

[10] World Health Organization. Executive Board 134 A69/17. Multisectoral action for a life course approach to healthy ageing: report by the Secretariat. 2016. Available: http://apps.who.int/gb/ebwha/pdf_files/EB134/B134_19-en.pdf. Accessed: 7 July 2016.

[11] Fortin M, Lapointe L, Hudon C, Vanasse A (2005). Multimorbidity is common to family practice: is it commonly researched?. Can Fam Physician.

[12] Ramond-Roquin A, Fortin M (2016). Towards increased visibility of multimorbidity research.. J Comorbidity..

[13] Marengoni A, Angleman S, Melis R, Mangialasche F, Karp A, Garmen A (2011). Aging with multimorbidity: a systematic review of the literature.. Ageing Res Rev.

[14] Chen CM (2006). CiteSpace II: Detecting and visualizing emerging trends and transient patterns in scientific literature.. JASIST.

[15] Salive ME (2013). Multimorbidity in older adults.. Epidemiol Rev.

[16] Bromet E, Andrade LH, Hwang I, Sampson NA, Alonso J, de Girolamo G (2011). Cross-national epidemiology of DSM-IV major depressive episode.. BMC Med.

[17] Adeloye D, Chua S, Lee CW, Basquill C, Papana A, Theodoratou E (2015). Global and regional estimates of COPD prevalence: systematic review and meta-analysis.. J Glob Health.

[18] Prados-Torres A, Calderon-Larranaga A, Hancco-Saavedra J, Poblador-Plou B, van den Akker M (2014). Multimorbidity patterns: a systematic review.. J Clin Epidemiol.

[19] Jackson CA, Jones M, Tooth L, Mishra GD, Byles J, Dobson A (2015). Multimorbidity patterns are differentially associated with functional ability and decline in a longitudinal cohort of older women.. Age Ageing.

[20] World Health Organization. Projections of mortality and causes of death, 2015 and 2030. Available: http://www.who.int/healthinfo/global_burden_disease/projections/en/. Accessed 10 July 2016.

[21] The World Bank. World Development Indicators. Available: http://data.worldbank.org/indicator/NY.GNP.PCAP.CD. Accessed: 7 July 2016.

[22] The World Bank. World Development Indicators. Available: http://data.worldbank.org/indicator/SP.DYN.LE00.IN. Accessed 7 July 2016.

[23] Menotti A, Mulder I, Nissinen A, Giampaoli S, Feskens EJ, Kromhout D (2001). Prevalence of morbidity and multimorbidity in elderly male populations and their impact on 10-year all-cause mortality: The FINE study (Finland, Italy, Netherlands, Elderly).. J Clin Epidemiol.

[24] Fortin M, Bravo G, Hudon C, Vanasse A, Lapointe L (2005). Prevalence of multimorbidity among adults seen in family practice.. Ann Fam Med.

[25] Wolff JL, Starfield B, Anderson G (2002). Prevalence, expenditures, and complications of multiple chronic conditions in the elderly.. Arch Intern Med.

[26] de Groot V, Beckerman H, Lankhorst GJ, Bouter LM (2003). How to measure comorbidity: a critical review of available methods.. J Clin Epidemiol.

[27] Fried LP Ferrucci L Darer J Williamson JD Anderson G Untangling the concepts of disability, frailty, and comorbidity: Implications for improved targeting and care. J Gerontol a-Biol. 2004;59:255-63. 10.1093/gerona/59.3.m255 15031310

[28] Krueger RF, Markon KE (2006). Reinterpreting comorbidity: A model-based approach to understanding and classifying psychopathology.. Annu Rev Clin Psychol.

[29] Vogeli C, Shields AE, Lee TA, Gibson TB, Marder WD, Weiss KB (2007). Multiple chronic conditions: Prevalence, health consequences, and implications for quality, care management, and costs.. J Gen Intern Med.

[30] Britt HC, Harrison CM, Miller GC, Knox SA (2008). Prevalence and patterns of multimorbidity in Australia.. Med J Aust.

[31] Schäfer I, von Leitner EC, Schon G, Koller D, Hansen H, Kolonko T (2010). Multimorbidity patterns in the elderly: a new approach of disease clustering identifies complex interrelations between chronic conditions.. PLoS One.

[32] Valderas JM, Starfield B, Sibbald B, Salisbury C, Roland M (2009). Defining comorbidity: implications for understanding health and health services.. Ann Fam Med.

[33] Deeks SG, Lewin SR, Havlir DV (2013). The end of AIDS: HIV infection as a chronic disease.. Lancet.

[34] Edwards BK, Noone AM, Mariotto AB, Simard EP, Boscoe FP, Henley SJ (2014). Annual Report to the Nation on the status of cancer, 1975-2010, featuring prevalence of comorbidity and impact on survival among persons with lung, colorectal, breast, or prostate cancer.. Cancer.

[35] Cesari M Vellas B Hsu FC Newman AB Doss H King AC A Physical activity intervention to treat the Frailty Syndrome in older persons-results from the LIFE-P study. J Gerontol a-Biol. 2015;70:216-22. 10.1093/gerona/glu099 PMC4311184 25387728

[36] Wehling M (2016). How to use the FORTA (“Fit fOR The Aged”) list to improve pharmacotherapy in the elderly.. Drug Res (Stuttg).

[37] Duarte MB, Rego MAV (2007). Depression and clinical illness: comorbidity in a geriatric outpatient clinic.. Cad Saude Publica.

[38] Wong SY, Mercer SW, Woo J, Leung J (2008). The influence of multi-morbidity and self-reported socio-economic standing on the prevalence of depression in an elderly Hong Kong population.. BMC Public Health.

[39] Thanapop S, Pannarunothai S, Chongsuvivatwong V (2009). Profile of hospital charges for chronic conditions by health status and severity level: a case study of 4 provinces in Thailand.. Asia Pac J Public Health.

[40] Gao X, Hofman A, Hu Y, Lin HD, Zhu CW, Jeekel J (2010). The Shanghai Changfeng Study: a community-based prospective cohort study of chronic diseases among middle-aged and elderly: objectives and design.. Eur J Epidemiol.

[41] de Souza Santos Machado V, Valadares AL, da Costa-Paiva LS, Moraes SS, Pinto-Neto AM (2012). Multimorbidity and associated factors in Brazilian women aged 40 to 65 years: a population-based study.. Menopause.

[42] Habib RR, Hojeij S, Elzein K, Chaaban J, Seyfert K (2014). Associations between life conditions and multi-morbidity in marginalized populations: the case of Palestinian refugees.. Eur J Public Health.

[43] Alaba O, Chola L (2013). The social determinants of multimorbidity in South Africa.. Int J Equity Health.

[44] Jerliu N, Toci E, Burazeri G, Ramadani N, Brand H (2013). Prevalence and socioeconomic correlates of chronic morbidity among elderly people in Kosovo: a population-based survey.. BMC Geriatr.

[45] Mir APB (2013). Need for geriatric dentistry training programs in Iran.. J Dent Educ.

[46] Nimako BA, Baiden F, Sackey SO, Binka F (2013). Multimorbidity of chronic diseases among adult patients presenting to an inner-city clinic in Ghana.. Global Health.

[47] Baijal G, Gupta T, Hotwani C, Laskar SG, Budrukkar A, Murthy V (2012). Impact of comorbidity on therapeutic decision-making in head and neck cancer: audit from a comprehensive cancer center in India.. Head Neck.

[48] Demirchyan A, Khachadourian V, Armenian HK, Petrosyan V (2013). Short and long term determinants of incident multimorbidity in a cohort of 1988 earthquake survivors in Armenia.. Int J Equity Health.

[49] Banjare P, Pradhan J (2014). Socio-economic inequalities in the prevalence of multi-morbidity among the rural elderly in Bargarh District of Odisha (India).. PLoS One.

[50] Khabala KB, Edwards JK, Baruani B, Sirengo M, Musembi P, Kosgei RJ (2015). Medication Adherence Clubs: a potential solution to managing large numbers of stable patients with multiple chronic diseases in informal settlements.. Trop Med Int Health.

[51] Mateen FJ, Kanters S, Kalyesubula R, Mukasa B, Kawuma E, Kengne AP (2013). Hypertension prevalence and Framingham risk score stratification in a large HIV-positive cohort in Uganda.. J Hypertens.

[52] Hien H, Berthe A, Drabo MK, Meda N, Konate B, Tou F (2014). Prevalence and patterns of multimorbidity among the elderly in Burkina Faso: cross-sectional study.. Trop Med Int Health.

[53] Wandera SO, Golaz V, Kwagala B, Ntozi J (2015). Factors associated with self-reported ill health among older Ugandans: a cross sectional study.. Arch Gerontol Geriatr.

[54] Jadad AR, To MJ, Emara M, Jones J (2011). Consideration of multiple chronic diseases in randomized controlled trials.. JAMA.

[55] van Weel C, Schellevis FG (2006). Comorbidity and guidelines: conflicting interests.. Lancet.

[56] Kowal P, Arokiasamy P, Afshar S, Pati S, Snodgrass JJ (2015). Multimorbidity: health care that counts “past one” for 1.2 billion older adults.. Lancet.

[57] Atun R (2015). Transitioning health systems for multimorbidity.. Lancet.

[58] Banerjee S (2015). Multimorbidity-older adults need health care that can count past one.. Lancet.

[59] Tinetti ME, Fried TR, Boyd CM (2012). Designing health care for the most common chronic condition–multimorbidity.. JAMA.

[60] Di Angelantonio E, Kaptoge S, Wormser D, Willeit P, Butterworth AS, Emerging Risk Factors Collaboration (2015). Association of cardiometabolic multimorbidity with mortality.. JAMA.

[61] Guthrie B, Payne K, Alderson P, McMurdo ME, Mercer SW (2012). Adapting clinical guidelines to take account of multimorbidity.. BMJ.

[62] Chetty R, Stepner M, Abraham S, Lin S, Scuderi B, Turner N (2016). The association between income and life expectancy in the United States, 2001-2014.. JAMA.

[63] Coventry P, Lovell K, Dickens C, Bower P, Chew-Graham C, McElvenny D (2015). Integrated primary care for patients with mental and physical multimorbidity: cluster randomised controlled trial of collaborative care for patients with depression comorbid with diabetes or cardiovascular disease.. BMJ.

[64] Navickas R, Petric V-K, Feigl AB, Seychell M (2016). Multimorbidity: what do we know? What should we do?. J Comorbidity..

[65] Vasan A, Ellner A, Lawn SD, Gupta N, Anatole M, Drobac P (2013). Strengthening of primary-care delivery in the developing world: IMAI and the need for integrated models of care.. Lancet Glob Health.

[66] Pearce N, Ebrahim S, McKee M, Lamptey P, Barreto ML, Matheson D (2014). The road to 25x25: how can the five-target strategy reach its goal?. Lancet Glob Health.

[67] Lefèvre T, d’Ivernois JF, De Andrade V, Crozet C, Lombrail P, Gagnayre R (2014). What do we mean by multimorbidity? An analysis of the literature on multimorbidity measures, associated factors, and impact on health services organization.. Rev Epidemiol Sante Publique.

