# Supplementary material for: Mapping the global research landscape and knowledge gaps on multimorbidity: a bibliometric study
Source: J Glob Health. 2017 Jun 10;7(1):010414. doi: 10.7189/jogh.07.010414 (PMC5475311; doi:10.7189/jogh.07.010414)
Supplement: Online Supplementary Document [file jogh-07-010414-s001.pdf]

Online Supplementary Document

Xu et al. Mapping the global research landscape and knowledge gaps on multimorbidity: a bibliometric study

J Glob Health 2017;7:010414

Contents

Appendix 1. Search strategies used for the study 2
Appendix 2. Percentage of the publications type, 1974-2016 3
Appendix 3. Percentage of publications language, 1974-2016 4
Appendix 4. Top 20 journals ranked by total number of publications, 1974-2016 5
Appendix 5. Top 20 authors ranked by total number of publications, 1974 - 2016 7
Appendix 6. Top 20 institutions ranked by the number of publications, 1974-2016 8
Appendix 7. Visualization of the institutions performance and collaborative networks, 1974-2016 9
Appendix 8. Top 15 funding agencies ranked by total number of publications, 1974-2016 ... 10
Appendix 9. Top 20 subject categories ranked by total number of publications, 1974-2016 . 11
Appendix 10. Visualization of subject categories and their connections, 1974-2016 12
Appendix 11. Full results- total number of publications, year of the first publication between 1974 and 2016, and country category by the World Bank, WHO region, and GDP/GNP in 2015 13
Appendix 12. Share of publications by World Bank (left) and WHO region (right), 1974-2016 16
Appendix 13. Characteristics of featured publications from different countries, 2001-2016 .. 17
Appendix 14: Top 15 most cited articles, 1974-2016 21

Appendix 1. Search strategies used for the study

No.	Search terms	Publications
#1	Multimorbidity OR multi-morbidity	2, 105
#2	multiple chronic diseases OR multiple chronic conditions	839
#3	Polymorbidity	64
#4	Polypathology	62
#5	Pluripathology	12
#6	Multipathology	3
#7	#1 OR #2 OR #3 OR #4 OR #5 OR #6	2, 864

The “Topic” search strategy was performed, which encompasses the title, abstract, keyword with the search terms.

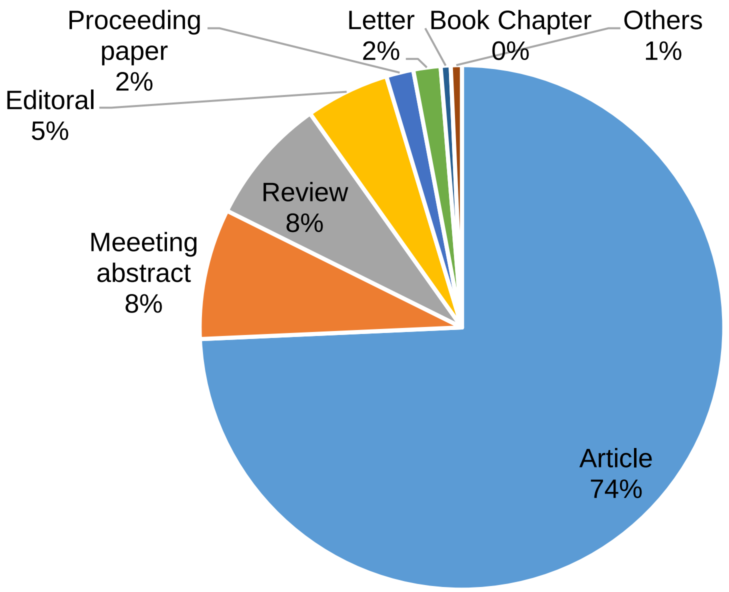

Appendix 2. Percentage of the publications type, 1974-2016

Appendix 3. Percentage of publications language, 1974-2016

Rank	Language	Number of publications	Percentage
1	English	2,490	86.9%
2	German	286	10.0%
3	Spanish	36	1.3%
4	French	30	1.0%
5	Russian	12	0.4%
6	Portuguese	3	0.1%
7	Slovenian	2	0.1%
8	Italian	1	0.0%
9	Icelandic	1	0.0%
10	Hungarian	1	0.0%
11	Dutch	1	0.0%
12	Czech	1	0.0%
	**Total**	**2,864**	**100.0%**

Appendix 4. Top 20 journals ranked by total number of publications, 1974-2016

Rank	Journal Name	Publications (%)	Journal category	Impact Factors (2015)
1	Journal of the American Geriatrics Society	74 (2.6)	Geriatrics and Gerontology	3.842
1	PLOS ONE	74 (2.6)	Science & Technology - Other Topics	3.057
3	BMC Family Practice	67 (2.3)	General & Internal Medicine	1.641
4	Gerontologist	58 (2.0)	Geriatrics & Gerontology	3.168
5	BMC Health Services Research	57 (2.0)	Health Care Sciences & Services	1.606
6	Zeitschrift für Gerontologie und Geriatrie	48 (1.7)	Geriatrics & Gerontology	0.769
7	Journal of General Internal Medicine	45 (1.6)	Health Care Sciences & Services; General & Internal Medicine	3.494
8	BMJ Open	42 (1.5)	General & Internal Medicine	2.562
9	Annals of Family Medicine	41 (1.4)	General & Internal Medicine	5.087
10	European Journal of Internal Medicine	38 (1.3)	General & Internal Medicine	2.591
11	BMC Public Health	37 (1.3)	Public, Environmental & Occupational Health	2.209
12	Medical Care	36 (1.3)	Health Care Sciences & Services; Public, Environmental & Occupational Health	3.081
13	Journal of Clinical Epidemiology	33 (1.2)	Health Care Sciences & Services; Public, Environmental & Occupational Health	4.703
14	Preventing Chronic Disease	32(1.1)	Public, Environmental & Occupational Health	2.170
15	BMC Geriatrics	28 (1.0)	Geriatrics & Gerontology	2.371
15	Archives of Gerontology and Geriatrics	28 (1.0)	Geriatrics & Gerontology	1.971
17	British Journal of General Practice	26 (1.0)	General & Internal Medicine	2.741
18	International Journal of Integrated Care	22 (0.8)	Health Policy & Services	1.557
19	Family Practice	20 (0.8)	General & Internal Medicine	2.022
20	Value in Health	19 (0.7)	Economics, Health Care Sciences & Services, Health Policy & Services	3.824

20	Journal of the American Medical Directors Association	19 (0.7)	Geriatrics & Gerontology	6.616
20	BMJ	19 (0.7)	Medicine, General & Internal	19.697
20	Age and Ageing	19 (0.7)	Geriatrics & Gerontology	4.201

Appendix 5. Top 15 authors ranked by total number of publications, 1974 - 2016

Rank	Authors	No. of publications	No. of citations	Country	Affiliation
1	Mercer SW	35	2136	UK	University of Glasgow
2	Boyd CM	35	676	USA	Johns Hopkins University
3	Marengoni A	34	737	Italy	University of Brescia
4	Fortin M	32	1431	Canada	Université de Sherbrooke
5	van den Bussche H	27	377	Germany	University of Hamburg
6	van den Akker M	24	918	Netherland	Maastricht University
7	Valderas JM	23	865	UK	University of Exeter
8	Guthrie B	23	375	UK	University of Dundee
9	Gensichen J	22	344	Germany	University of Jena
10	Wurm S	21	113	Germany	University of Erlangen-Nuremberg
11	Bower P	21	220	UK	University of Manchester
12	Tinetti ME	20	510	USA	
13	Scherer M	20	153	Germany	Johannes Gutenberg Univ Mainz
14	Salisbury C	20	707	UK	Yale University
15	Hudon C	20	1212	Canada	Université de Sherbrooke

Appendix 6. Top 20 institutions ranked by the number of publications, 1974-2016

Rank	Institution	No. of publications	Country
1	Johns Hopkins University	97	USA
2	Harvard University	81	USA
3	University of California System	77	USA
4	Ruprecht Karl University Heidelberg	66	Germany
5	University of Toronto	63	Canada
6	Free University of Berlin	61	Germany
7	University of Michigan System	56	USA
8	University of Glasgow	56	UK
9	Karolinska Institutet	55	Sweden
10	Yale University	54	USA
11	University of London	54	UK
12	University of Manchester	52	UK
13	University of Michigan	51	USA
14	Maastricht University	50	Netherland
15	Radboud University Nijmegen	49	Netherland
16	University of Washington Seattle	48	USA
17	University of Washington	48	USA
18	University of Hamburg	47	Germany
19	Us Department of Veteran Affairs	45	USA
20	University of Sydney	44	Australia

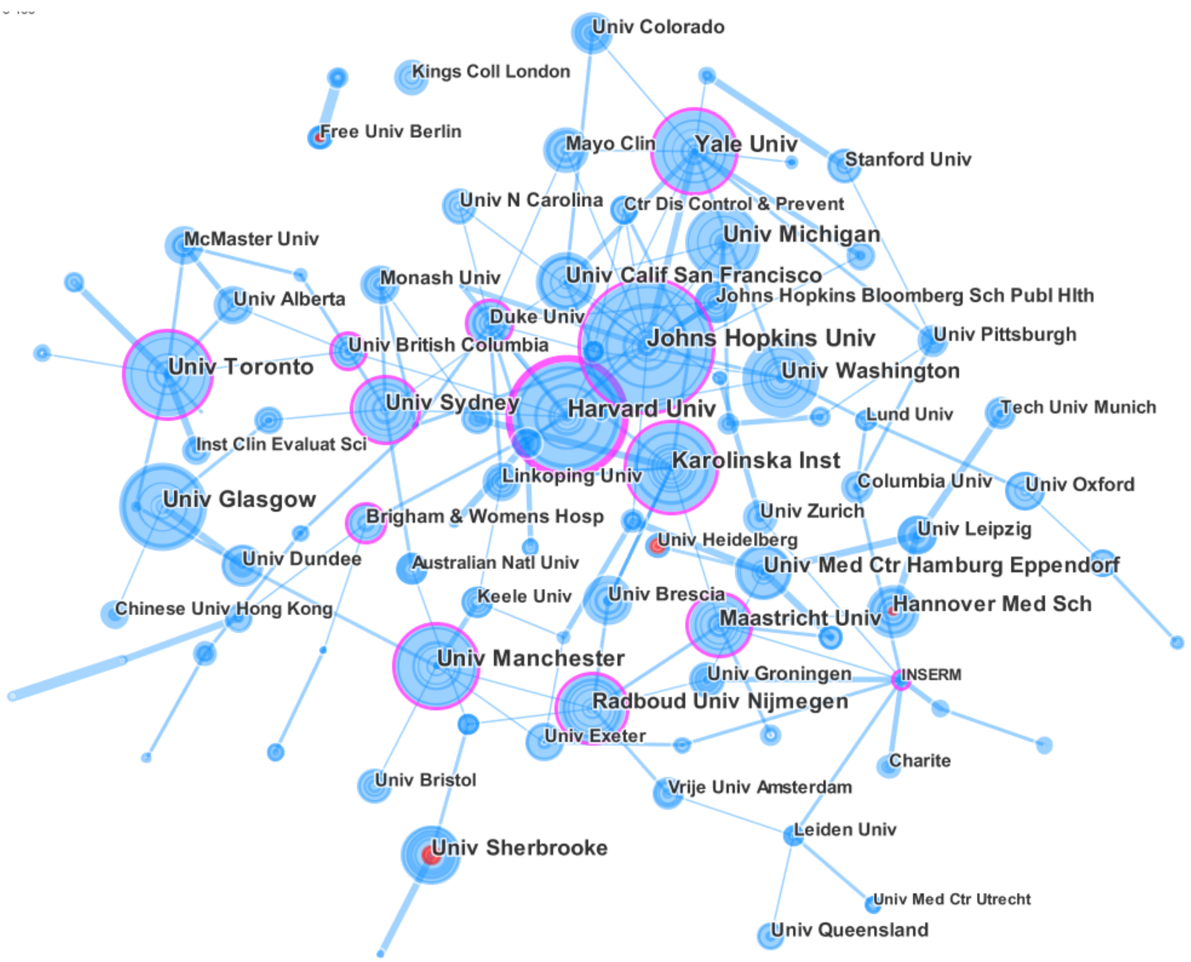

Appendix 7. Visualization of the institutions performance and collaborative networks, 1974-2016

Note: The color of a ring indicated the time of a corresponding citation. The thickness of a ring was proportional to the number of citations in one year time slicing. Purple rims of nodes indicate the importance of nodes in terms of betweenness centrality (≥0.1). The red ring indicates that a citation burst is detected in the corresponding time slice. The betweenness centrality of a node quantifies the importance of the node's position in a network. A node of high centrality is usually one that connects two or more large groups of nodes with the node itself in-between. Centrality scores are normalized to the unit interval of [0, 1].

Appendix 8. Top 15 funding agencies ranked by total number of publications, 1974-2016

Rank	Funding agencies	Countries	No. of publications
1	National Institute on Aging	USA	77
2	German Federal Ministry of Education and Research	Germany	53
3	National Institutes of Health	USA	42
4	Canadian Institutes of Health Research	Canada	39
5	Agency for Healthcare Research and Quality	USA	38
6	European Union/European Commission/European Community	Europe	33
7	National Health and Medical Research Council	Australia	28
8	Pfizer	USA	26
9	John A. Hartford Foundation	USA	21
10	National Heart Lung and Blood Institute	USA	16
11	The Atlantic Philanthropies	USA	13
12	GlaxoSmithKline	UK	12
13	Starr Foundation	USA	11
14	National Institute for Health Research	UK	11
15	National Institute of Mental Health	USA	10

Appendix 9. Top 20 subject categories ranked by total number of publications, 1974-2016

Rank	Subject category	No. of publications
1	Medicine General Internal	691
2	Health Care Sciences Services	422
3	Geriatrics Gerontology	416
4	Public Environmental Occupational Health	381
5	Gerontology	292
6	Health Policy Services	236
7	Primary Health Care	209
8	Psychiatry	154
9	Pharmacology Pharmacy	115
10	Cardiac Cardiovascular Systems	84
11	Multidisciplinary Sciences	79
12	Clinical Neurology	78
13	Nursing	73
14	Oncology	52
15	Psychology Clinical	50
16	Medical Informatics	47
17	Respiratory System	43
18	Surgery	42
19	Endocrinology Metabolism	42
20	Psychology	40

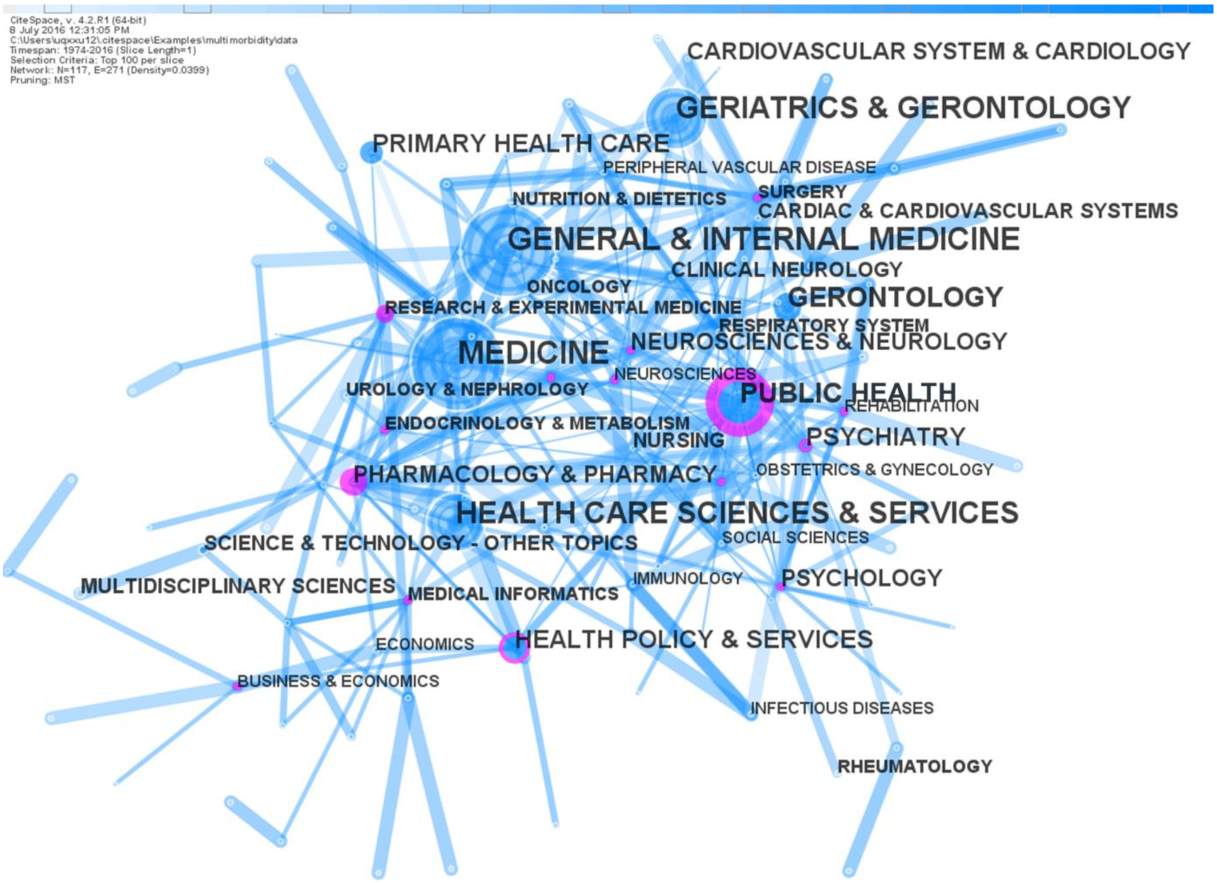

Appendix 10. Visualization of subject categories and their connections, 1974-2016

This figure maps the relationship among the different subject categories. The color of a ring indicated the time of a corresponding citation. The thickness of a ring was proportional to the number of citations in one year time slicing. Purple rims of nodes indicate the importance of nodes in terms of betweenness centrality (≥0.1). The betweenness centrality of a node quantifies the importance of the node's position in a network. A node of high centrality is usually one that connects two or more large groups of nodes with the node itself in-between. Centrality scores are normalized to the unit interval of [0, 1].

Appendix 11. Full results- total number of publications, year of the first publication between 1974 and 2016, and country category by the World Bank, WHO region, and GDP/GNP in 2015

Country Name	No. of publication	Year of first publication	Country category	WHO region category	Country Code	2015 GDP	2015 GNP
Albania	1	2013	UM	Europe	ALB	11	4290
Argentina	2	2013	H	Americas	ARG	548	13640
Armenia	1	2013	LM	Europe	ARM	11	3880
Australia	184	2005	H	West Pacific	AUS	1340	60070
Austria	57	1992	H	Europe	AUT	374	47120
Bangladesh	1	2011	LM	South-East Asia	BGD	195	1190
Barbados	2	2015	H	Americas	BRB	4	14800
Belgium	52	1998	H	Europe	BEL	454	44360
Bosnia and Herzegovina	4	2013	UM	Europe	BIH	16	4680
Botswana	1	2015	UM	Africa	BWA	14	6510
Brazil	25	2005	UM	Americas	BRA	1775	9850
Bulgaria	3	2015	UM	Europe	BGR	49	7220
Burkina Faso	2	2014	L	Africa	BFA	11	660
Canada	219	2001	H	Americas	CAN	1551	47500
Chile	1	2009	H	Americas	CHL	240	14060
China	52	2008	UM	West Pacific	CHN	10866	7820
Colombia	2	2014	UM	Americas	COL	292	7130
Croatia	9	2009	H	Europe	HRV	49	12690
Czech Republic	13	2003	H	Europe	CZE	182	18050
Denmark	40	1992	H	Europe	DNK	295	58590
Eritrea	1	2014	L	Africa	ERI	3	480
Estonia	2	1991	H	Europe	EST	23	18480
Ethiopia	1	2016	L	Africa	ETH	62	590
Finland	19	1994	H	Europe	FIN	230	46360
France	93	1990	H	Europe	FRA	2422	40580
Germany	511	1974	H	Europe	DEU	3356	45790
Ghana	2	2013	LM	Africa	GHA	38	1480
Greece	16	2010	H	Europe	GRC	195	20290
Hungary	8	2010	H	Europe	HUN	121	12990

Iceland	6	1998	H	Europe	ISL	17	49730
India	20	2012	LM	South-East Asia	IND	2074	1590
Iran, Islamic Rep.	4	2013	UM	Eastern Mediterranean	IRN	425	6550
Ireland	69	2007	H	Europe	IRL	238	46680
Israel	11	2006	H	Europe	ISR	296	35440
Italy	142	1998	H	Europe	ITA	1815	32790
Japan	13	2004	H	West Pacific	JPN	4123	36680
Kenya	3	2015	LM	Africa	KEN	63	1340
Korea, Rep.	14	2007	H	West Pacific	KOR	1378	27440
Lebanon	1	2014	UM	Eastern Mediterranean	LBN	47	7930
Lesotho	1	2016	LM	Africa	LSO	2	1330
Lithuania	4	2012	H	Europe	LTU	41	15000
Luxembourg	4	2013	H	Europe	LUX	58	77000
Malawi	1	2013	L	Africa	MWI	7	350
Malaysia	1	2010	UM	South-East Asia	MYS	296	10570
Malta	2	2010	H	Europe	MLT	10	21000
Mexico	5	2014	UM	Americas	MEX	1144	9710
Morocco	3	2013	LM	Eastern Mediterranean	MAR	100	3040
Netherlands	208	1998	H	Europe	NLD	753	48940
New Zealand	17	2010	H	West Pacific	NZL	174	40080
Nigeria	1	2015	LM	Africa	NGA	481	2820
Norway	34	1997	H	Europe	NOR	388	93820
Pakistan	2	2012	LM	Eastern Mediterranean	PAK	270	1440
Panama	1	2016	UM	Americas	PAN	52	12050
Philippines	1	2015	LM	West Pacific	PHL	292	3540
Poland	24	2011	H	Europe	POL	475	13370
Portugal	12	2007	H	Europe	PRT	199	20530
Qatar	1	2015	H	Eastern Mediterranean	QAT	167	85430
Romania	4	2013	UM	Europe	ROU	178	9500
Russian Federation	12	1999	H	Europe	RUS	1326	11400

Saudi Arabia	2	2012	H	Eastern Mediterranean	SAU	646	23550
Serbia	4	2013	UM	Europe	SRB	37	5500
Singapore	5	2013	H	West Pacific	SGP	293	52090
Slovak Republic	5	2009	H	Europe	SVK	87	17310
Slovenia	12	2000	H	Europe	SVN	43	22610
South Africa	11	2010	UM	Africa	ZAF	313	6050
Spain	168	1990	H	Europe	ESP	1199	28520
Sweden	109	1997	H	Europe	SWE	493	57810
Switzerland	100	1985	H	Europe	CHE	665	84180
Taiwan	23	2004	H	West Pacific	TWN	524	23390
Thailand	2	2009	UM	South-East Asia	THA	395	5620
Turkey	10	2010	UM	Europe	TUR	718	9950
Uganda	3	2013	L	Africa	UGA	26	670
United Arab Emirates	1	2016	H	Eastern Mediterranean	ARE	370	43170
United Kingdom	389	1991	H	Europe	GBR	2849	43340
United States	895	1983	H	Americas	USA	17947	54960
Vietnam	2	2015	LM	West Pacific	VNM	194	1980

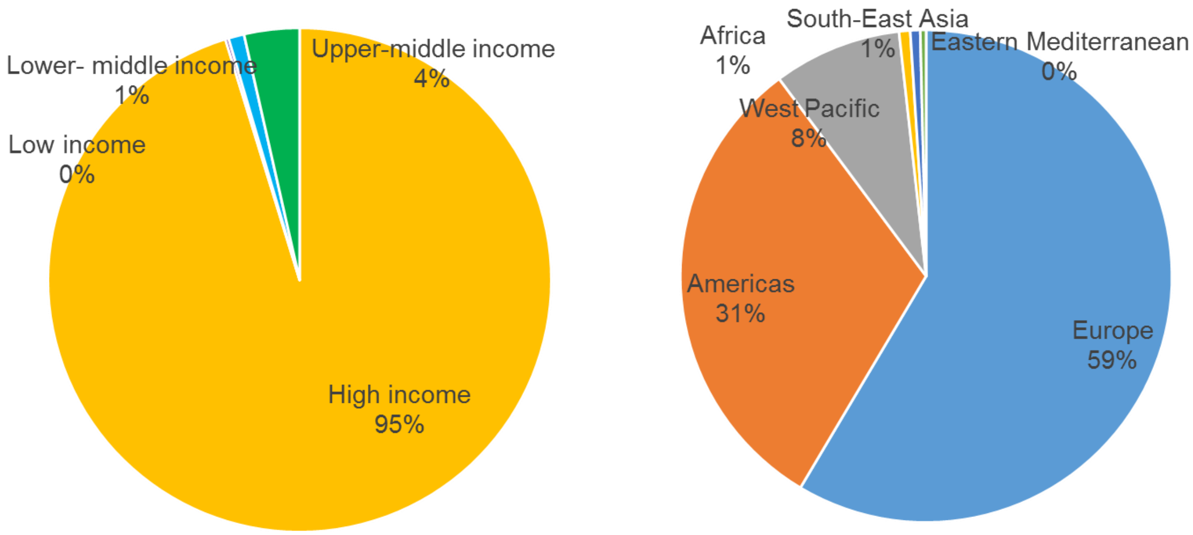

Appendix 12. Share of publications by World Bank (left) and WHO region (right), 1974-2016

Appendix 13. Characteristics of featured publications from different countries, 2001-2016

No.	Country of origin	WHO Regions	Year	Title	Citations (n)	Collaborations	Learn from HIs
	**High income countries**						
1	Finland, Italy, Netherlands	Europe	2001	Prevalence of morbidity and multimorbidity in elderly male populations and their impact on 10-year all-cause mortality: The FINE study (Finland, Italy, Netherlands, Elderly)[1]	80	HIs	-
2	USA	Americas	2002	Prevalence, expenditures, and complications of multiple chronic conditions in the elderly[2]	737	-	-
3	Netherlands	Europe	2003	How to measure comorbidity: a critical review of available methods[3]	646	-	-
4	USA	Americas	2004	Untangling the concepts of disability, frailty, and comorbidity: Implications for improved targeting and care[4]	942	-	-
5	Canada	Americas	2005	Prevalence of multimorbidity among adults seen in family practice[5]	322	-	-
6	USA	Americas	2006	Reinterpreting comorbidity: A model-based approach to understanding and classifying psychopathology[6]	373	-	-
7	USA	Americas	2007	Multiple chronic conditions: Prevalence, health consequences, and implications for quality, care management, and costs[7]	249	-	-
8	Australia	West Pacific	2008	Prevalence and patterns of multimorbidity in Australia[8]	129	--	-
9	UK, USA	Europe Americas	2009	Defining Comorbidity: Implications for Understanding Health and Health Services[9]	279	HIs	-
10	Germany	Europe	2010	Multimorbidity Patterns in the Elderly: A New Approach of Disease Clustering Identifies Complex Interrelations between Chronic Conditions[10]	82	-	-

11	Italy, Sweden, Netherlands	Europe	2011	Aging with multimorbidity: A systematic review of the literature[11]	269	HIs	-
12	UK	Europe	2012	Epidemiology of multimorbidity and implications for health care, research, and medical education: a cross-sectional study[12]	633	-	-
13	USA, Australia	Americas, West Pacific	2013	The end of AIDS: HIV infection as a chronic disease[13]	166	HIs	-
14	USA	Americas	2014	Annual Report to the Nation on the status of cancer, 1975-2010, featuring prevalence of comorbidity and impact on survival among persons with lung, colorectal, breast, or prostate cancer[14]	160	-	-
15	France, USA	Europe Americas	2015	A physical activity intervention to treat the frailty syndrome in older persons-results from the LIFE-P study[15]	20	HIs	-
16	Germany	Europe	2016	How to Use the FORTA ("Fit fOR The Aged") List to Improve Pharmacotherapy in the Elderly[16]	3	-	-
	**Upper-middle income countries**						
1	Brazil	Americas	2007	Depression and clinical illness: comorbidity in a geriatric outpatient clinic[17]	14	-	Y
2	China (Hong Kong)	West Pacific	2008	The influence of multi-morbidity and self-reported socio-economic standing on the prevalence of depression in an elderly Hong Kong population[18]	19	-	Y
3	Thailand	West Pacific	2009	Profile of hospital charges for chronic conditions by health status and severity level: a case study of 4 provinces in Thailand[19]	1	-	Y
4	China	West Pacific	2010	The Shanghai Changfeng Study: a community-based prospective cohort study of chronic diseases among middle-aged and elderly: objectives and design[20]	19	-	Y
5	Brazil	Americas	2012	Multimorbidity and associated factors in Brazilian women aged 40 to 65 years: a population-based study[21]	13	-	Y

6	South Africa	Africa	2013	The social determinants of multimorbidity in South Africa[22]	14	-	Y
7	Serbia	Europe	2013	Prevalence and socioeconomic correlates of chronic morbidity among elderly people in Kosovo: a population-based survey[23]	6	-	Y
8	Iran	Eastern Mediterranean	2013	Need for Geriatric Dentistry Training Programs in Iran[24]	1	-	Y
9	China	West Pacific	2014	Epidemiology of multimorbidity in China and implications for the healthcare system: cross-sectional survey among 162,464 community household residents in southern China[25]	22	-	Y
10	Lebanon	Eastern Mediterranean	2014	Associations between life conditions and multi-morbidity in marginalized populations: the case of Palestinian refugees[26]	3	-	Y
11	Tukey	Europe	2014	Evaluation of potentially inappropriate drug use and medical non-adherence in a community-dwelling elderly population: A cross-sectional study[27]	1	-	Y
	**Lower-middle income countries**						
1	Bangladesh	South-East Asia	2011	Prevalence and Patterns of Multimorbidity among Elderly People in Rural Bangladesh: A Cross-sectional Study[28]	24	-	Y
2	India	South-East Asia	2012	Impact of comorbidity on therapeutic decision-making in head and neck cancer: Audit from a comprehensive cancer center in India[29]	6	-	Y
3	Armenia	Europe	2013	Short and long term determinants of incident multimorbidity in a cohort of 1988 earthquake survivors in Armenia[30]	1	-	Y
4	Ghana	Africa	2014	Multimorbidity of chronic diseases among adult patients presenting to an inner-city clinic in Ghana[31]	6	-	Y
5	India	South-East Asia	2014	Socio-Economic Inequalities in the Prevalence of Multi-Morbidity among the Rural Elderly in Bargarh District of Odisha (India)[32]	4	-	Y

6	Kenya	Africa	2015	Medication Adherence Clubs: a potential solution to managing large numbers of stable patients with multiple chronic diseases in informal settlements[33]	1	-	Y
7	China, Ghana, India, Mexico, Russia and South Africa	West Pacific, Africa, South-East Asia, Americas, Europe	2015	The impact of multimorbidity on adult physical and mental health in low- and middle-income countries: what does the study on global ageing and adult health (SAGE) reveal?[34]	4	HIs, UMIs, LMIs, LIs	Y
	**Low income countries**						
1	Uganda	Africa	2013	Hypertension prevalence and Framingham risk score stratification in a large HIV-positive cohort in Uganda[35]	15	-	Y
2	Uganda	Africa	2015	Factors associated with self-reported ill health among older Ugandans: a cross sectional study[36]	0	-	Y
3	Burkina Faso	Africa	2014	Prevalence and patterns of multimorbidity among the elderly in Burkina Faso: cross-sectional study[37]	0	-	Y

Appendix 14: Top 15 most cited articles, 1974-2016

No	Title of articles	Year	Journal	Authors	Total Citations
1	Untangling the concepts of disability, frailty, and comorbidity: Implications for improved targeting and care	2004	Journals of Gerontology Series A-Biological Sciences And Medical Sciences	Fried, LP; Ferrucci, L; Darer, J; *et al*.	941
2	Prevalence, expenditures, and complications of multiple chronic conditions in the elderly	2002	Archives of Internal Medicine	Wolff, JL; Starfield, B; Anderson, G	735
3	How to measure comorbidity: a critical review of available methods	2003	Journal of Clinical Epidemiology	de Groot, V; Beckerman, H; Lankhorst, GJ; *et al*.	644
4	Epidemiology of multimorbidity and implications for health care, research, and medical education: a cross-sectional study	2012	Lancet	Barnett, Karen; Mercer, Stewart W.; Norbury, Michael; *et al*.	631
5	2013 ACC/AHA Guideline on the Treatment of Blood Cholesterol to Reduce Atherosclerotic Cardiovascular Risk in Adults A Report of the American College of Cardiology/American Heart Association Task Force on Practice Guidelines	2014	Circulation	Stone, Neil J.; Robinson, Jennifer G.; Lichtenstein, Alice H.; *et al*.	605
6	2013 ACC/AHA Guideline on the Treatment of Blood Cholesterol to Reduce Atherosclerotic Cardiovascular Risk in Adults A Report of the American College of Cardiology/American Heart Association Task Force on Practice Guidelines	2014	Journal of The American College of Cardiology	Stone, Neil J.; Robinson, Jennifer G.; Lichtenstein, Alice H.; *et al*.	558
7	Reinterpreting comorbidity: A model-based approach to understanding and classifying psychopathology	2006	Annual Review of Clinical Psychology	Krueger, Robert E.; Markon, Kristian E.	371
8	Multimorbidity in general practice: Prevalence, incidence, and determinants of co-occurring chronic and recurrent diseases	1998	Journal of Clinical Epidemiology	van den Akker, M; Buntinx, F; Metsemakers, JFM; *et al*.	368
9	Health-related quality of life associated with chronic conditions in eight countries: Results from the International Quality of Life Assessment (IQOLA) Project	2004	Quality of Life Research	Alonso, J; Ferrer, M; Gandek, B; *et al*.	345
10	Prevalence of multimorbidity among adults seen in family practice	2005	Annals of Family Medicine	Fortin, M; Bravo, G; Hudon, C; *et al*.	321

11	Self-management education programs in chronic disease - A systematic review and methodological critique of the literature	2004	Archives of Internal Medicine	Warsi, A; Wang, PS; LaValley, MP; *et al*.	305
12	Defining Comorbidity: Implications for Understanding Health and Health Services	2009	Annals of Family Medicine	Valderas, Jose M.; Starfield, Barbara; Sibbald, Bonnie; *et al*.	279
13	Aging with multimorbidity: A systematic review of the literature	2011	Ageing Research Reviews	Marengoni, Alessandra; Angleman, Sara; Melis, Rene; *et al*.	266
14	Multiple chronic conditions: Prevalence, health consequences, and implications for quality, care management, and costs	2007	Journal of General Internal Medicine	Vogeli, Christine; Shields, Alexandra E.; Lee, Todd A.; *et al*.	249
15	Household food insufficiency is associated with poorer health	2003	Journal of Nutrition	Vozoris, NT; Tarasuk, VS	204

1. Menotti A, Mulder I, Nissinen A, Giampaoli S, Feskens EJ, Kromhout D. Prevalence of morbidity and multimorbidity in elderly male populations and their impact on 10-year all-cause mortality: The FINE study (Finland, Italy, Netherlands, Elderly). J Clin Epidemiol. 2001; **54**(7): 680-6.
2. Wolff JL, Starfield B, Anderson G. Prevalence, expenditures, and complications of multiple chronic conditions in the elderly. Archives of internal medicine. 2002; **162**(20): 2269-76.
3. de Groot V, Beckerman H, Lankhorst GJ, Bouter LM. How to measure comorbidity: a critical review of available methods. J Clin Epidemiol. 2003; **56**(3): 221-9.
4. Fried LP, Ferrucci L, Darer J, Williamson JD, Anderson G. Untangling the concepts of disability, frailty, and comorbidity: Implications for improved targeting and care. J Gerontol a-Biol. 2004; **59**(3): 255-63.
5. Fortin M, Bravo G, Hudon C, Vanasse A, Lapointe L. Prevalence of multimorbidity among adults seen in family practice. Ann Fam Med. 2005; **3**(3): 223-8.
6. Krueger RE, Markon KE. Reinterpreting comorbidity: A model-based approach to understanding and classifying psychopathology. Annu Rev Clin Psycho. 2006; **2**: 111-33.
7. Vogeli C, Shields AE, Lee TA, Gibson TB, Marder WD, Weiss KB, et al. Multiple chronic conditions: Prevalence, health consequences, and implications for quality, care management, and costs. Journal of General Internal Medicine. 2007; **22**: 391-5.
8. Britt HC, Harrison CM, Miller GC, Knox SA. Prevalence and patterns of multimorbidity in Australia. Med J Aust. 2008; **189**(2): 72-7.

9. Valderas JM, Starfield B, Sibbald B, Salisbury C, Roland M. Defining Comorbidity: Implications for Understanding Health and Health Services. Annals of Family Medicine. 2009; **7**(4): 357-63.
10. Schafer I, von Leitner EC, Schon G, Koller D, Hansen H, Kolonko T, et al. Multimorbidity patterns in the elderly: a new approach of disease clustering identifies complex interrelations between chronic conditions. Plos One. 2010; **5**(12): e15941.
11. Marengoni A, Angleman S, Melis R, Mangialasche F, Karp A, Garmen A, et al. Aging with multimorbidity: a systematic review of the literature. Ageing Res Rev. 2011; **10**(4): 430-9.
12. Barnett K, Mercer SW, Norbury M, Watt G, Wyke S, Guthrie B. Epidemiology of multimorbidity and implications for health care, research, and medical education: a cross-sectional study. Lancet. 2012; **380**(9836): 37-43.
13. Deeks SG, Lewin SR, Havlir DV. The end of AIDS: HIV infection as a chronic disease. Lancet. 2013; **382**(9903): 1525-33.
14. Edwards BK, Noone AM, Mariotto AB, Simard EP, Boscoe FP, Henley SJ, et al. Annual Report to the Nation on the status of cancer, 1975-2010, featuring prevalence of comorbidity and impact on survival among persons with lung, colorectal, breast, or prostate cancer. Cancer. 2014; **120**(9): 1290-314.
15. Cesari M, Vellas B, Hsu FC, Newman AB, Doss H, King AC, et al. A Physical Activity Intervention to Treat the Frailty Syndrome in Older Persons-Results From the LIFE-P Study. J Gerontol a-Biol. 2015; **70**(2): 216-22.
16. Wehling M. How to Use the FORTA ("Fit fOR The Aged") List to Improve Pharmacotherapy in the Elderly. Drug Res. 2016; **66**(2): 57-62.
17. Duarte MB, Rego MAV. Depression and clinical illness: comorbidity in a geriatric outpatient clinic. Cad Saude Publica. 2007; **23**(3): 691-700.
18. Wong SY, Mercer SW, Woo J, Leung J. The influence of multi-morbidity and self-reported socio-economic standing on the prevalence of depression in an elderly Hong Kong population. BMC public health. 2008; **8**: 119.
19. Thanapop S, Pannarunothai S, Chongsuvivatwong V. Profile of Hospital Charges for Chronic Conditions by Health Status and Severity Level: A Case Study of 4 Provinces in Thailand. Asia-Pac J Public He. 2009; **21**(2): 196-204.
20. Gao X, Hofman A, Hu Y, Lin HD, Zhu CW, Jeekel J, et al. The Shanghai Changfeng Study: a community-based prospective cohort study of chronic diseases among middle-aged and elderly: objectives and design. Eur J Epidemiol. 2010; **25**(12): 885-93.
21. de Souza Santos Machado V, Valadares AL, da Costa-Paiva LS, Moraes SS, Pinto-Neto AM. Multimorbidity and associated factors in Brazilian women aged 40 to 65 years: a population-based study. Menopause. 2012; **19**(5): 569-75.
22. Alaba O, Chola L. The social determinants of multimorbidity in South Africa. Int J Equity Health. 2013; **12**: 63.
23. Jerliu N, Toci E, Burazeri G, Ramadani N, Brand H. Prevalence and socioeconomic correlates of chronic morbidity among elderly people in Kosovo: a population-based survey. Bmc Geriatrics. 2013; **13**.
24. Mir APB. Need for Geriatric Dentistry Training Programs in Iran. J Dent Educ. 2013; **77**(1): 113-7.
25. Wang HH, Wang JJ, Wong SY, Wong MC, Li FJ, Wang PX, et al. Epidemiology of multimorbidity in China and implications for the healthcare system: cross-sectional survey among 162,464 community household residents in southern China. BMC Med. 2014; **12**: 188.
26. Habib RR, Hojeij S, Elzein K, Chaaban J, Seyfert K. Associations between life conditions and multi-morbidity in marginalized populations: the case of Palestinian refugees. Eur J Public Health. 2014; **24**(5): 727-33.
27. Sonmez Y, Asci H, Izmirli GO, Gundogar D, Cankara FN, Yesilot S. Evaluation of Potentially Inappropriate Drug Use and Medical Non-Adherence in a Community-Dwelling Elderly Population: A Cross-Sectional Study. Turk J Geriatr. 2014; **17**(2): 125-33.

28. Khanam MA, Streatfield PK, Kabir ZN, Qiu C, Cornelius C, Wahlin A. Prevalence and patterns of multimorbidity among elderly people in rural Bangladesh: a cross-sectional study. J Health Popul Nutr. 2011; **29**(4): 406-14.
29. Baijal G, Gupta T, Hotwani C, Laskar SG, Budrukkar A, Murthy V, et al. Impact of comorbidity on therapeutic decision-making in head and neck cancer: Audit from a comprehensive cancer center in India. Head Neck-J Sci Spec. 2012; **34**(9): 1251-4.
30. Demirchyan A, Khachadourian V, Armenian HK, Petrosyan V. Short and long term determinants of incident multimorbidity in a cohort of 1988 earthquake survivors in Armenia. Int J Equity Health. 2013; **12**: 68.
31. Nimako BA, Baiden F, Sackey SO, Binka F. Multimorbidity of chronic diseases among adult patients presenting to an inner-city clinic in Ghana. Globalization and health. 2013; **9**: 61.
32. Banjare P, Pradhan J. Socio-Economic Inequalities in the Prevalence of Multi-Morbidity among the Rural Elderly in Bargarh District of Odisha (India). Plos One. 2014; **9**(6).
33. Khabala KB, Edwards JK, Baruani B, Sirengo M, Musembi P, Kosgei RJ, et al. Medication Adherence Clubs: a potential solution to managing large numbers of stable patients with multiple chronic diseases in informal settlements. Tropical Medicine & International Health. 2015; **20**(10): 1265-70.
34. Arokiasamy P, Uttamacharya U, Jain K, Biritwum RB, Yawson AE, Wu F, et al. The impact of multimorbidity on adult physical and mental health in low- and middle-income countries: what does the study on global ageing and adult health (SAGE) reveal? BMC Med. 2015; **13**: 178.
35. Mateen FJ, Kanters S, Kalyesubula R, Mukasa B, Kawuma E, Kengne AP, et al. Hypertension prevalence and Framingham risk score stratification in a large HIV-positive cohort in Uganda. Journal of hypertension. 2013; **31**(7): 1372-8.
36. Wandera SO, Golaz V, Kwagala B, Ntozi J. Factors associated with self-reported ill health among older Ugandans: A cross sectional study. Arch Gerontol Geriat. 2015; **61**(2): 231-9.
37. Hien H, Berthe A, Drabo MK, Meda N, Konate B, Tou F, et al. Prevalence and patterns of multimorbidity among the elderly in Burkina Faso: cross-sectional study. Trop Med Int Health. 2014; **19**(11): 1328-33.
